# Curcumin’s Radioprotective Effects on Zebrafish Embryos

**DOI:** 10.3390/antiox13111281

**Published:** 2024-10-23

**Authors:** Gaia Pucci, Gaetano Savoca, Giuseppina Iacoviello, Giorgio Russo, Giusi I. Forte, Vincenzo Cavalieri

**Affiliations:** 1 Institute of Bioimaging and Complex Biological Systems (IBSBC)—National Research Council (CNR), Cefalù Secondary Site, C/da Pietrapollastra-Pisciotto, 90015 Cefalù, Italy; gaiapucci@cnr.it (G.P.); giorgio-russo@cnr.it (G.R.); 2 Department of Biological, Chemical and Pharmaceutical Sciences and Technologies (STeBiCeF), University of Palermo, Viale delle Scienze Bld. 16, 90128 Palermo, Italy; 3 Radiation Oncology Unit, ARNAS-Civico Hospital, 90100 Palermo, Italy; gaetano.savoca@arnascivico.it (G.S.); giuseppina.iacoviello@arnascivico.it (G.I.); 4 Zebrafish Laboratory, Advanced Technologies Network (ATeN) Center, University of Palermo, Viale delle Scienze Bld. 16, 90128 Palermo, Italy

**Keywords:** zebrafish, curcumin, radioprotection, radiomodifier, radiation

## Abstract

Radiation modifiers are largely studied for their contribution to enlarging the treatment window. Curcumin is already known for its antioxidant properties; however, its role as a radioprotector in preclinical studies is affected by the well-known low absorption and bioavailability of curcumin. In this study, curcumin’s radioprotection ability has been evaluated in zebrafish larvae, by taking advantage of quantifying curcumin absorption and evaluating its fluorescence in transparent embryos. A curcumin range of 1–10 μM was tested to select the non-toxic concentrations to be used for a pre-treatment of photon beam irradiation using a 2–15 Gy range of doses. The post-treatment analysis within 120 h post-fertilization (hpf) included an assessment of mortality and malformation rates and behavioral and gene expression analysis. A total of 2.5 and 5 μM of curcumin pre-treatment showed a radioprotective role, significantly reducing the frequency of embryo malformations and damaged entities. This sparing effect disappeared using 15 Gy, showing the radiation effect’s prevalence. Gene expression analysis reconducted this radioprotective ability for antioxidant gene network activation. The curcumin-induced activation of the antioxidant gene network promoted radioprotection in zebrafish.

## 1. Introduction

Conventional radiotherapy (RT) protocols are based on the administration of photon beams in the form of low Linear Energy Transfer (LET) radiations (X-rays, γ-rays), which deposit a relatively small amount of energy on the target and disperse it to the surrounding healthy tissue, due to scattering phenomena. RT treatment plans aim to fully control tumor growth, minimizing dosage absorption of normal tissues and organs at risk, to prevent adverse effects and toxicity [1,2].

Conventional RT produces a high incidence of dermatitis, pneumonitis, cataracts, neurocognitive impairment, myelosuppression, and mucositis/enteritis [3], and about 90% of patients experience acute skin toxicities [4], chronic symptoms, or severe organ dysfunctions that can produce a decrease in quality of life [5], leading to the inability to administer the intended therapy. In particular, the radiation-induced damage to normal tissues may vary depending on the type and volume of the irradiated organs, individual radiosensitivity, type of radiation (photons, electrons, and charged particles), total dose, and dose per fraction delivered [6,7,8,9].

Significant improvements in the precision of dose delivery to tumor targets have been gained by the constant advancements in treatment modalities and image-guided RT technologies [8]. However, it is still important to broaden the therapeutic window between normal tissue complications and tumor suppression.

In this study, a wide range of compounds have been tested as radiomodifiers in combination with RT schedules, to find drugs able to enhance tumor sensitivity to ionizing radiation (IR) or to be radioprotective for normal tissues [10]. Some bio-active compounds isolated from natural sources can minimize the collateral effects of oncological patients, thanks to their antioxidants and immune-modulating properties, thus providing benefits as radioprotectors [11,12]. Among these compounds, curcumin [(1E,6E)-1,7-bis(4-hydroxy-3-methoxyphenyl)-1,6-heptadiene-3,5-dione] has been largely studied for its pleiotropic medicinal and antineoplastic properties, due to its ability to interact with numerous targets such as kinases, adhesion molecules, transcription factors, growth factors, and pro-inflammatory cytokines [13]. In addition, numerous in vitro and preclinical studies have described its dual role of radioprotector and radiosensitizer in association with IR, through mechanisms summarized as follows: (1) the direct enhancement of the tumoricidal effect, (2) the reversion of radioresistance, by reducing the pro-survival response mechanisms of cancer cells, and (3) the alleviation of toxicity, by antioxidant and anti-inflammatory properties [10,14,15].

Some preclinical studies have already highlighted its role in reducing IR-induced toxicity, such as mucositis, dermatitis, and other organ-specific injuries [16,17]. However, the full comprehension of curcumin’s radioprotective potential is strongly affected by the well-known low water solubility, low absorption, and bioavailability [18]. Indeed, after ingestion, curcumin is metabolized by reduction and conjugation. However, the resulting metabolites show reduced biological activities compared to curcumin [19]. Its high metabolism rate makes it an unstable, reactive, and unavailable compound with poor pharmacokinetic/pharmacodynamic properties [20].

Thus, considering its possible clinical use, curcumin needs to be stringently analyzed in appropriate models and conditions of radiation injury. Among animals used for these experimental purposes, the zebrafish (*Danio rerio*) is a validated vertebrate model for diseases, drug screening, target identification, and pharmacology [21,22]. Among the main advantages of using this model are the fast response time, easy maintenance, transparency for easy observation, high fecundity, and rapid generation time, which permit it to reach high statistical validity [23]. In addition, the genome comparison revealed 72% of similarity and 84% of homologous genes [24]. Furthermore, in the contest of radiobiology research, the zebrafish has become a milestone for the screening of different radiation beams and modifiers in a complex organism and for convenient manipulation conditions (small adult individuals 2–3 cm long, a wide tolerance of maintenance and transportation, and unnecessary sterility) [25,26,27]. 

Embryo development is ex utero and extremely rapid: all major organs and tissues are fully developed within 48 hours post-fertilization (hpf), and this allows us to carry out a very detailed post-treatment phenotypic analysis in a very small time window [28]. In addition, embryos are completely transparent until 72 hpf, and this makes it possible to study their real-time development and possible alterations as well as to make xenograft tumor models. During organogenesis, zebrafish embryos are freely permeable to water and a wide range of electrolytes, cryoprotectants, drugs, small molecules, peptides, and dyes, providing easy access for the administration of compounds, which can be directly administered into the fish water [29,30]. As can be imagined, embryogenesis is a very radiosensitive stage of the life cycle, due to continuous cell division and the presence of an aqueous environment, which leads to a homogeneous dose distribution [31]. Thus, in the contest of the radiobiology literature, the irradiation of zebrafish embryos has become a method to evaluate IR-induced toxicity, as they produce well-known alterations that can be followed and quantified during the embryogenesis steps and by dedicated scoring scales [27,32,33,34].

Thus, zebrafish embryos represent a valid system for screening radiomodifiers. This study aimed to evaluate curcumin as a radioprotective molecule in zebrafish embryos and larvae. Our results showed that curcumin pre-treatment at the doses of 2.5 and 5 μM exerts a radioprotective effect, significantly reducing the occurrence of embryo malformation induced by photon beam irradiation in the range of 2–10 Gy. This sparing effect disappeared using 15 Gy, showing the prevalence of the IR dose effect. The radioprotective effect has been associated with the upregulation of markers involved in the antioxidant gene network.

## 2. Materials and Methods

### 2.1. Zebrafish Care and Mating

The Zebrafish Laboratory of the Advanced Technologies Network (ATeN) Center at the University of Palermo is authorized to use zebrafish (*Danio rerio*) for scientific research by the Italian Ministry of Health (aut. prot. no. 24/2023-UT of 13 June 2023). Wild-type (AB strain) zebrafish adults were acquired from the European Zebrafish Resource Center (Karlsruhe Institute of Technology, Eggenstein-Leopoldshafen, Germany) and kept in a recirculating aquaculture system (Tecniplast, West Chester, PA, USA) under standard conditions (14 h light/10 h dark cycle, temperature 28 °C, conductivity 500 ± 50 µS, and PH 7.5), according to National (Italian D.lgs 26/2014) and European (2010/63/EU) animal welfare laws. Breeding and embryo manipulation were performed as described in [35].

All the experiments here described were performed on embryos and larvae within 120 hpf, not subjected to animal experimentation rules according to the above-mentioned laws.

### 2.2. Embryo Treatments 

**Figure 1:**
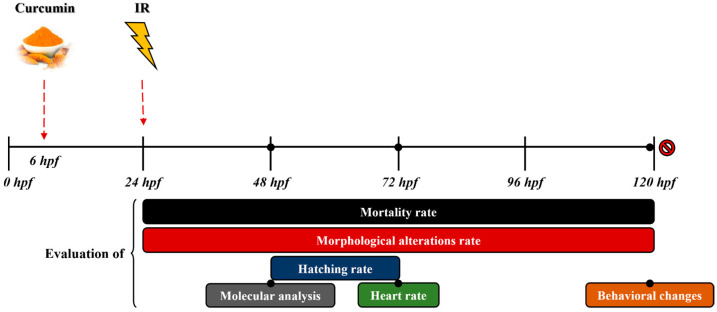
A synoptic diagram of the experimental plan. The evaluation of the mortality and morphological alteration rate was completed from 24 to 120 hpf, while the assessment of the hatching rate was completed at 48 and 72 hpf. The molecular analysis, heart rate evaluation, and behavioral assays were performed at 48, 72, and 119 hpf, respectively.

Experiments were performed on viable, normal, and synchronous embryos. For each type of treatment, a group of 15–20 embryos was sorted as 1 embryo/well of a standard 96-well polystyrene microplate (SPL life sciences, Pocheon-si, Republic of Korea) in a 200 µL embryo medium (E3) and kept at 28 °C. The E3 was changed daily to avoid contamination and infection spreading. A synoptic diagram of the experimental treatment timing and follow-up steps is reported in Figure 1.

### 2.3. Curcumin Preparation, Treatment, and Detection

A 10 mM curcumin stock solution was prepared by dissolving the powder (Merck, Darmstadt, Germany) in Dimethyl sulfoxide (DMSO) (Euroclone, Pero, Italy). Then, the final concentration treatment solutions (1, 2.5, 5, 7.5, and 10 μM) were properly diluted in E3 and subjected to sonication (TRANSSONIC T310, Elma—ultrasonic effective power approx. 35 W) to facilitate its solubilization. Each experiment included control groups of sibling embryos exposed to standard E3 medium and to the equivalent volume of DMSO (up to 0.1%).

Taking advantage of the curcumin fluorescent property [36,37], it was possible to monitor its absorption and accumulation in the embryo body by microscope observations. Thus, accumulation and signal decay tests were performed, considering a parallel set of untreated synchronous embryos as the control of the baseline fluorescence.

In particular, embryos were incubated at 6, 9, and 22 hpf with 0, 1, 2.5, 5, 7.5, and 10 μM curcumin and then observed at 24 hpf using a Multidimensional Fluorescence Stereomicroscope M205 FA (Leica Microsystems, Wetzlar, Germany). Images were captured by a DFC 550 camera (Leica Microsystems), using LAS X Software version 5.2.2 (Leica Microsystems). Then, images were analyzed by using ImageJ software version 1.54k to evaluate the relative increase in fluorescence intensity at increasing curcumin concentrations, with respect to untreated controls. In addition, to evaluate the signal decay, embryos were treated with 5 μM curcumin at 6, 9, and 22 hpf. Then, 24 hpf embryos were washed three times in E3 at room temperature (rt), and image acquisitions occurred every 30–40 min, until the complete loss of the fluorescence signal.

Finally, considering the decay time window, showing a progressive reduction in the curcumin auto-fluorescence signal in the embryos, a continuous 1–10 µM curcumin treatment was scheduled, refreshing the treated E3 medium two times/day from 6 to 120 hpf, to assess the curcumin-induced toxicity.

### 2.4. Radiation Setting and Treatment

The IR treatment (X-rays) was performed at 24 hpf, at rt. The Siemens Primus clinical linear accelerator (Siemens Medical Systems, Concord, CA, USA) was used, releasing photon rays of 6 MV nominal energy. The Linac was calibrated according to the reference conditions defined by the International Atomic Energy Agency Technical Reports Series No. 398 “Determination of absorbed dose in external beam radiotherapy” [38]. The irradiation setup and dose distribution were determined using the Pinnacle treatment planning system (Philips Medical Systems, Andover MA, USA). The X-ray treatment was performed using doses of 2, 4, 6, 8, 10, and 15 Gy, at a dose rate of 2 Gy/min and a standard LET range of 0.22–0.37 keV/μm at 2 cm from the beam peak. The plates, surrounded by bolus bags (15 cm × 15 cm × 1.5 cm), were positioned between polymethyl methacrylate and CORK slabs, to avoid the sparing effect and to assure a homogeneous radiation exposure. The isocenter was positioned in the plate’s geometrical center. 

### 2.5. Combined Treatment

A total of 6 hpf embryos were subjected to 18 h pre-treatment with 2.5 and 5 μM curcumin and then subjected to X-rays at 24 hpf, using the doses of 2, 4, 8, 10, and 15 Gy. After IR treatment, the E3 medium was refreshed, and the day after, the morphological analysis started.

### 2.6. Survival and Morphological Analysis

The mortality assessment was confirmed by the absence of a heartbeat, blood circulation, or spontaneous movements within the chorion. It started at 24 hpf and was evaluated every 24 h up to 120 hpf. Survival was calculated as a percentage of viable embryos to the total number of treated embryos for each experimental condition [39].

The search for typical malformations, such as a partial or total absence of pigmentation (PIGM), Spinal Curvature (SC), pericardial edema (PE), and the inhibition of yolk sac resorption (Yolk Malabsorption, YM), started at 48 hpf and continued every 24 h up to 120 hpf [40]. Morphology was visually assessed by microscopy and was photo-documented daily, after anesthesia with 50 mg/L of tricaine methanesulfonate (MS-222 Sigma-Aldrich, St. Louis, MO, USA). Digital images were acquired for each time point, and the ImageJ software (https://imagej.nih.gov/ij/) was used to quantify morphometric parameters, including the larval body length, head length, eye length, pericardial edema, and yolk sac diameter, in accordance with [34].

The PE size values were used to calculate the Protection Rate (PR) parameter at 10 Gy, by using the following formula: [1 − (PE measurement in combined treatment/PE measurement in IR treatment) × 100].

### 2.7. Hatching and Heart Rate Detection

The hatching rate (HR) was evaluated, at 48 and 72 hpf. The hatched embryo percentage was calculated with respect to the total number of embryos × 100 [41].

To measure heart rate, fish larvae at 72 hpf were acclimatized to rt and anesthetized by soaking in 168 mg/L buffered tricaine methanesulfonate and examined under a DMi8 inverted microscope (Leica Microsystems, Milan, Italy). Individual videos, for a range of 9–15 randomly selected embryos from each experimental group, were recorded and heartbeats in 60 s (bpm) were manually counted three times, by two distinct operators for each larva.

### 2.8. Behavioral Analysis

The locomotor behavior of the control and treated larvae was assessed at 119 hpf, by positioning each 96-well plate, containing larvae, into the Zebralab video-tracking platform (ViewPoint Behavior Technology, Lyon, France). After acclimatization for 15 min at 50% light illumination, the larvae were positioned in the testing chamber. Larvae activity levels were recorded for 30 min, to evaluate the swim speed and the outlined movement, by evaluating the route type, i.e., short routes through small movements or long routes through large movements. The video output was analyzed with the ZebraLab Tracking Mode v3.22.3.89 (ViewPoint Behavior Technology, Lyon, France), and the raw data were processed with ViewPoint FastData Manager v2.4.0.2510 (ViewPoint Behavior Technology, Lyon, France). The locomotor behavior analysis was performed three times, at the same time of the day, using independent batches of larvae.

### 2.9. RNA Extraction, Reverse Transcription, and Real-Time Quantitative PCR

The total RNA from batches of 20 control and treated embryos at 48 hpf was extracted using the RNeasy Plus Mini kit (Qiagen, Milan, Italy). The quality and quantity of RNA were analyzed by a spectrophotometer and agarose gel electrophoresis, as described in [42]. cDNA synthesis was performed by the High-Capacity cDNA Reverse Transcription Kit (Applied Biosystems, Life Technologies Italia, ThermoFisher Scientific, Monza, Italy), according to the manufacturer’s protocol. A total of 10 ng of the resulting cDNA were used as the template for qPCR assays, based on previously reported methods [43,44,45]. The expression levels of the following genes were evaluated: *catalase (cat*), *superoxide dismutase 1* (*sod1*), *superoxide dismutase 2* (*sod2*), *glutathione peroxidase 1a* (*gpx1a*), *glutathione peroxidase 4a* (*gpx4a*)*, xanthine dehydrogenase* (*xdh*), *glutathione s-transferase pi* (*gstp*), *glutathione s-transferase pi 1a* (*gstp1a*), *lactate dehydrogenase a* (*ldha*), and *signal transducer and activator of transcription 3* (*stat 3*).

qPCR experiments were performed in triplicate on a StepOnePlus Real-Time PCR System (Applied Biosystems, ThermoFisher Scientific, Monza, Italy) using SYBRGreen detection chemistry. The oligonucleotide primers for *cat*, *sod2*, and *ldha* were described previously [35], while primers for the remaining genes are indicated in Table 1. In each experiment, a no-template control was included, and ROX was used as a reference of background fluorescence. Finally, a melting curve analysis was performed to check for any specificity. The *ribosomal protein L13* (*rpL13a*) mRNA was used as a housekeeping gene [35]. Relative quantification was performed using the comparative Ct method (∆∆Ct).

### 2.10. Statistical Analysis

A statistical data assessment was performed using GraphPad Instat (Version 3.05). Differences between observed and expected distributions between two groups of values were evaluated by using a contingency table (two rows, two columns) and a Fisher’s exact test, to calculate the *p* value (*p*) and the Odds ratio (OR) with a 95% Confidence interval (CI). This approach was used for the significance evaluation of malformation rates (%) at 96–120 hpf, in pre-treated embryos with 5 µM curcumin vs. the only irradiated ones for each dose (2–15 Gy), and to compare the SC or PE frequency at 96 hpf in pre-treated embryos vs. the only irradiated ones for the doses 10 and 15 Gy. 

On the other hand, a one-way analysis of variance (ANOVA) was used to analyze if variation among the variables’ means is significantly greater than expected by chance. In particular, it was applied to evaluate the significant variation in the morphometric parameter PE, obtained for pre-treated embryos vs. the only irradiated ones, at 72 and 96 hpf for each dose used (10–15 Gy). Overall, statistical significance was defined at *p* ≤ 0.05.

## 3. Results

### 3.1. The Evaluation of Curcumin Absorption and Toxicity on Zebrafish Embryos

**Figure 2:**
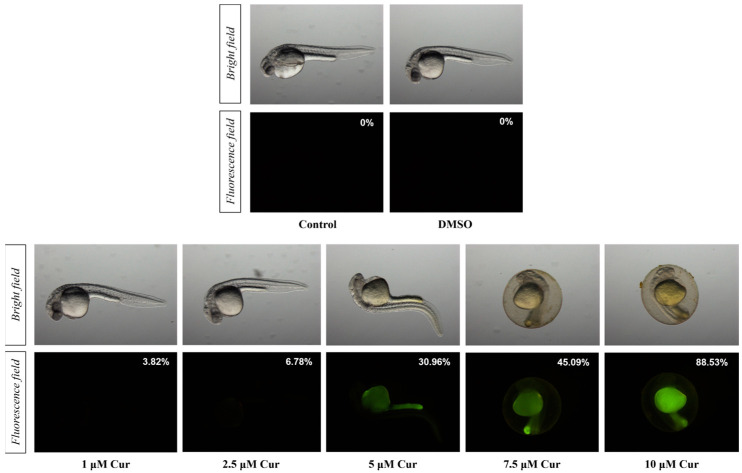
The evaluation of the curcumin auto-fluorescence signal (%) at 24 hpf in treated embryos with 1–10 μM curcumin (Cur), with respect to untreated controls.

Curcumin absorption was evaluated in 24 hpf embryos, quantifying the emitted fluorescence intensity in relation to the absorbed curcumin, administered at 6, 9, and 22 hpf in the 1–10 µM range of concentration. Fluorescence signal started to be detected using 1 µM curcumin and increased in a dose-dependent manner (Figure 2). Considering the prevalent area of the fluorescence signal, curcumin appeared to be accumulated in the yolk sac, whereas no background fluorescence signal was detected in the E3 medium containing curcumin, as previously reported [36]. 

The signal decay was evaluated by treating embryos with 5 μM curcumin at 6, 9, and 22 hpf. Then, 24 hpf embryos were observed until the complete loss of the fluorescence signal, showing complete signal decay in about 5 h (Table 2). Both the accumulation and decay tests confirmed about 30% of fluorescent signal in 24 hpf embryos treated with 5 μM curcumin with respect to untreated controls (Figure 2). 

**Figure 3:**
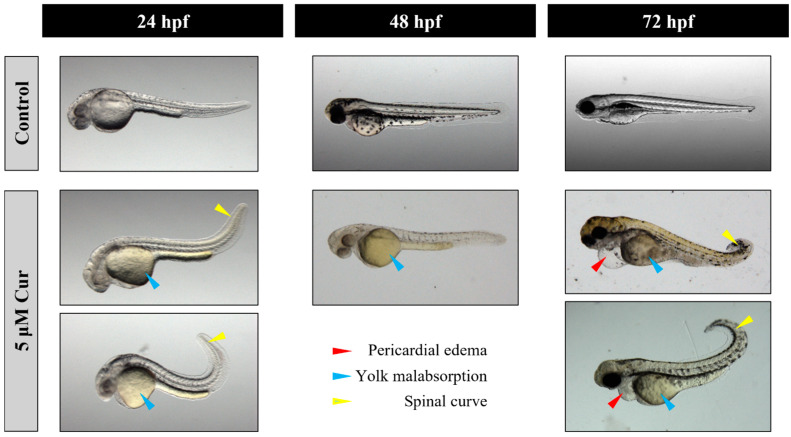
Representative images of the main malformations observed after treatment with 5 μM curcumin (Cur), such as YM (from 24 hpf), SC (from 24 hpf), PE (from 72 hpf), and PIGM (from 48 hpf). Pictures were taken in a light field.

Then, embryos were continuously exposed to the E3 medium containing curcumin in the range of 1–10 μM, which was replenished twice a day from 6 to 120 hpf, and the occurrence of mortality and developmental malformations were evaluated. Curcumin treatment inflicted gross malformations in a dose-dependent manner from 5 μM onwards, being 100% lethal at concentrations of 7.5 and 10 μM, at 72 and 48 hpf, respectively. Lower mortalities of 31%, 26%, and 46% were detected at 120 hpf for embryos exposed to 1, 2.5, and 5 μM, respectively (Figure S1). The number of embryos showing one or more morphological abnormalities appeared to be prevalent from 5 μM onwards, with values ranging from 12% to 47% at 24 and 120 hpf, respectively (Figure S1). Among the main alterations observed, SC prevailed, appearing from 24 hpf in embryos treated with 5 μM up to 72% at 120 hpf (Figure S2 and Figure 3). Fewer embryos appeared to be affected by SC malformations by the exposure to 1 and 2.5 μM curcumin, appearing at 48 hpf up to 120 hpf with a maximum frequency of 66%. PE was also observed, appearing at 48 hpf with a frequency of 40% following the 5 μM curcumin treatment (Figure S2 and Figure 3). The hatching rate analysis showed that the treatment with 5 μM curcumin led to a delay of this phenomenon at 48 hpf with respect to controls (37.5% vs. 93%), and then it recovered at 72 hpf (100% hatched embryos).

The behavioral analysis was also performed at 119 hpf in the alive embryos treated with 1–5 μM curcumin, using the ZebraBox platform (ViewPoint Behavior Technology), showing that curcumin did not induce significant alterations in the locomotor activity, evaluated as the mean swimming velocity of the treated embryos compared to controls. The observation of blood circulation, as well as the manual evaluation of the heart-beating rate, did not reveal any dysfunction in treated embryos compared to controls. 

### 3.2. Irradiation Treatment with Conventional X-Rays

The daily assessment of irradiated embryo viability, morphological alterations, and behavioral defects showed a correlation with the radiation dose administered. Data presented in Figures S3 and S4 are the mean of four experiments. The IR treatment led to very low mortality for embryos exposed to 2–15 Gy of X-rays with respect to the untreated ones. However, malformations appeared in a dose-dependent manner. Gross alterations were observed from 8 Gy onwards, with frequencies of 50% and 82% in 15 Gy-treated embryos at 48 and 120 hpf, respectively. Among the malformations observed, PE prevailed for the highest doses. It appeared at 48 hpf in embryos treated with 10 and 15 Gy, with the final percentages of 62% and 98% of affected specimens at 120 hpf, respectively. By contrast, fewer embryos appeared to be affected by PE following the exposure to doses lower than 8 Gy. SC was also observed, starting from 48 hpf in embryos treated with 8, 10, and 15 Gy (16%, 38%, and 49%, respectively) and at 72 hpf using lower doses. YM and PIGM were also found starting at 2 Gy, already at 48 hpf. 

Compared to untreated controls, the hatching rate of IR-treated embryos was delayed in a dose-dependent manner. Indeed, at 48 hpf, the rate of hatched embryos was 86% and 39% for 2 and 15 Gy, respectively, vs. 100% of controls. This delay was recovered at 72 hpf for doses lower than 6 Gy, but not for specimens treated with 8–15 Gy of IR.

The behavioral analysis of 119 hpf larvae showed that the IR treatment did not significantly affect the mean swimming velocity of IR-treated embryos, compared to controls. Instead, a difference was observed in the traveled distance. In particular, the irradiated embryos preferred to cross small distances rather than larger ones, with respect to the controls. For the dose range of 2–10 Gy, we observed an increase in the small distance values, ranging from +30.25 to +150.86 mm, and a reduction in the large distance values, ranging from −0.1 to −7.12 mm. 

The manual evaluation of the heart-beating rate revealed a small dose-dependent decrease (in the range from −6% to −22%) in treated embryos compared to controls. However, a slight increase was observed at 15 Gy (+9.8%).

### 3.3. Curcumin and X-Rays Combined Treatment

**Figure 4:**
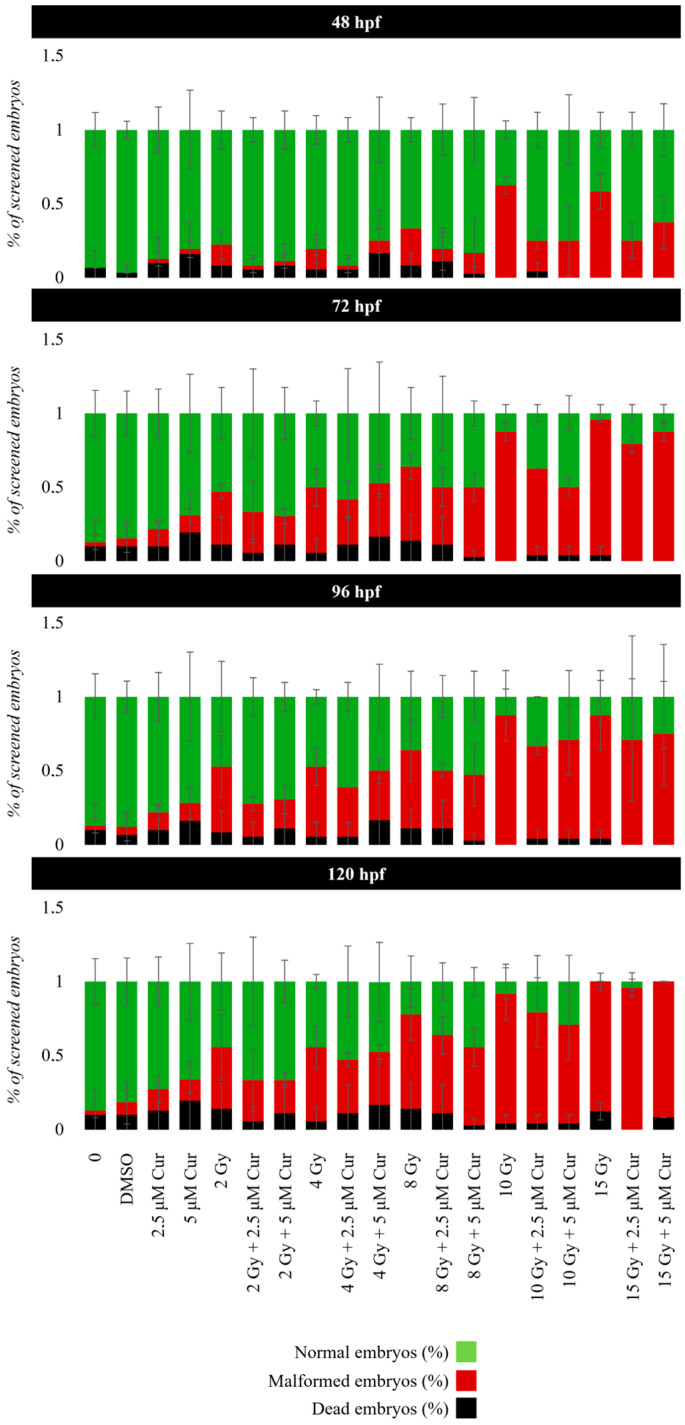
Normal (green bar), dead (black bar), and abnormal (red bar) embryo rates of developing zebrafish embryos exposed to a combination of curcumin (Cur) pre-treatment with concentrations of 2.5 or 5 μM, followed by irradiation with 0, 2, 4, 8, 10, or 15 Gy of X-rays. Data are presented as the mean of 3 experiments. Error bar = ±SD.

The concentrations of 2.5 and 5 µM were chosen to test the role of the curcumin pre-treatment in combination with the administration of 0–15 Gy doses of conventional X-rays. In this case, 6 hpf embryos were subjected to 18 h pre-treatment, instead of continuous treatment, before irradiation at 24 hpf (Figures S1 and S2). The 18 h pre-treatment without IR administration produced 14% of malformed embryos at 120 hpf with both curcumin concentrations, in comparison to 30 and 47% found with the 2.5 and 5 μM continuous treatments, respectively (Figures S1 and S2 and Figure 4). Furthermore, no embryos showed the most serious malformation PE at 120 hpf.

The embryo viability was not significantly affected by the combined treatment with both curcumin concentrations within 120 hpf, with respect to the controls. This was expected, considering the low mortality given by radiation treatment alone with the same dose range. Instead, a pronounced protective effect was observed, in terms of reduced percentages of malformed embryos, after a combined treatment using both the chosen curcumin concentrations, with respect to the only irradiated embryos (Figure 4). This sparing effect is visible as significantly reduced malformations, with the frequency in the 2–10 Gy range, and it is greater for a higher curcumin concentration (5 μM), as the following reports in 120 hpf embryos subjected to the combined treatment, with respect to the only irradiated ones:

2 Gy: 22% vs. 41% (*p*: 0.0017; OR: 0.3577; 95% CI: 0.1880 to 0.6808);

4 Gy: 35% vs. 49% (*p*: 0.2255; OR: 0.6687; 95% CI: 0.3678 to 1.216);

8 Gy: 52% vs. 63% (*p*: 0.0057; OR: 0.4127; 95% CI: 0.2198 to 0.7749);

10 Gy: 66% vs. 87% (*p*: 0.0002; OR: 0.2093; 95% CI: 0.08982 to 0.4876).

**Figure 5:**
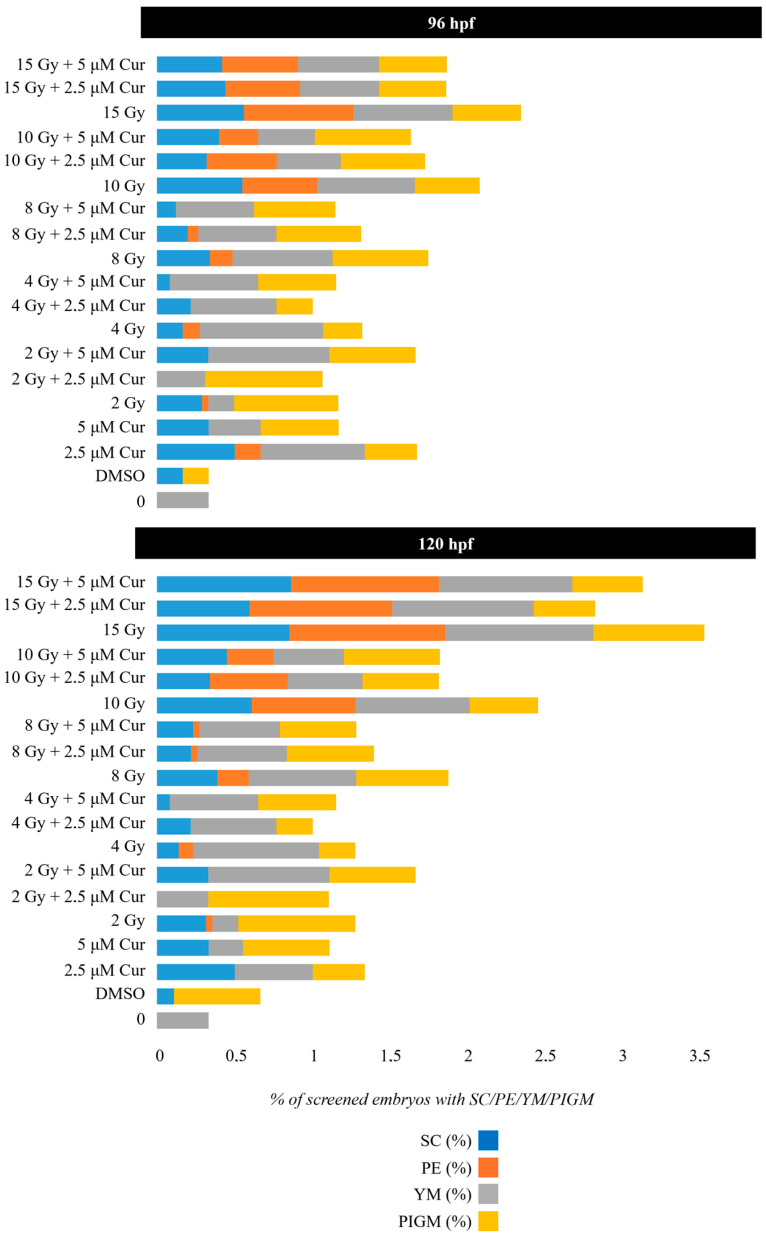
The distribution (%) of the main malformations observed in 96 and 120 hpf malformed zebrafish embryos exposed to a combination of curcumin (Cur) pre-treatment with concentrations of 2.5 or 5 μM, followed by irradiation with 0, 2, 4, 8, 10, or 15 Gy of X-rays: SC (blue bar), PE (orange bar), YM (gray bar), PIGM (yellow bar). Data are presented as the mean of 3 experiments.

**Figure 6:**
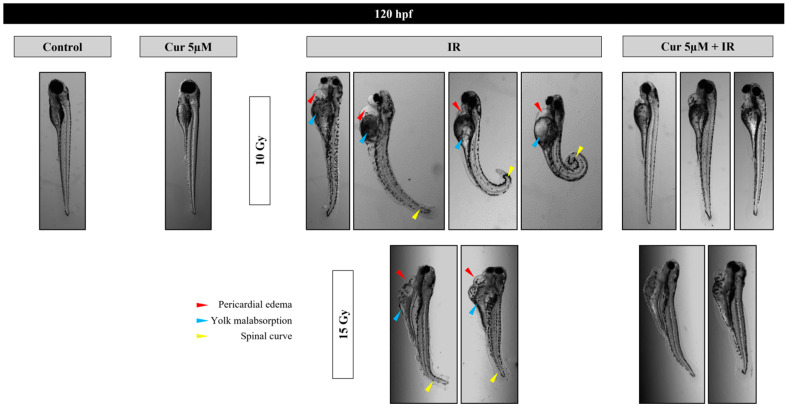
Representative images of the main malformations (PE, SC, YM) observed, at 120 hpf, after treatment with 10 Gy or 15 Gy alone or combined with both 2.5 and 5 μM curcumin (Cur) concentration, vs. controls.

**Figure 7:**
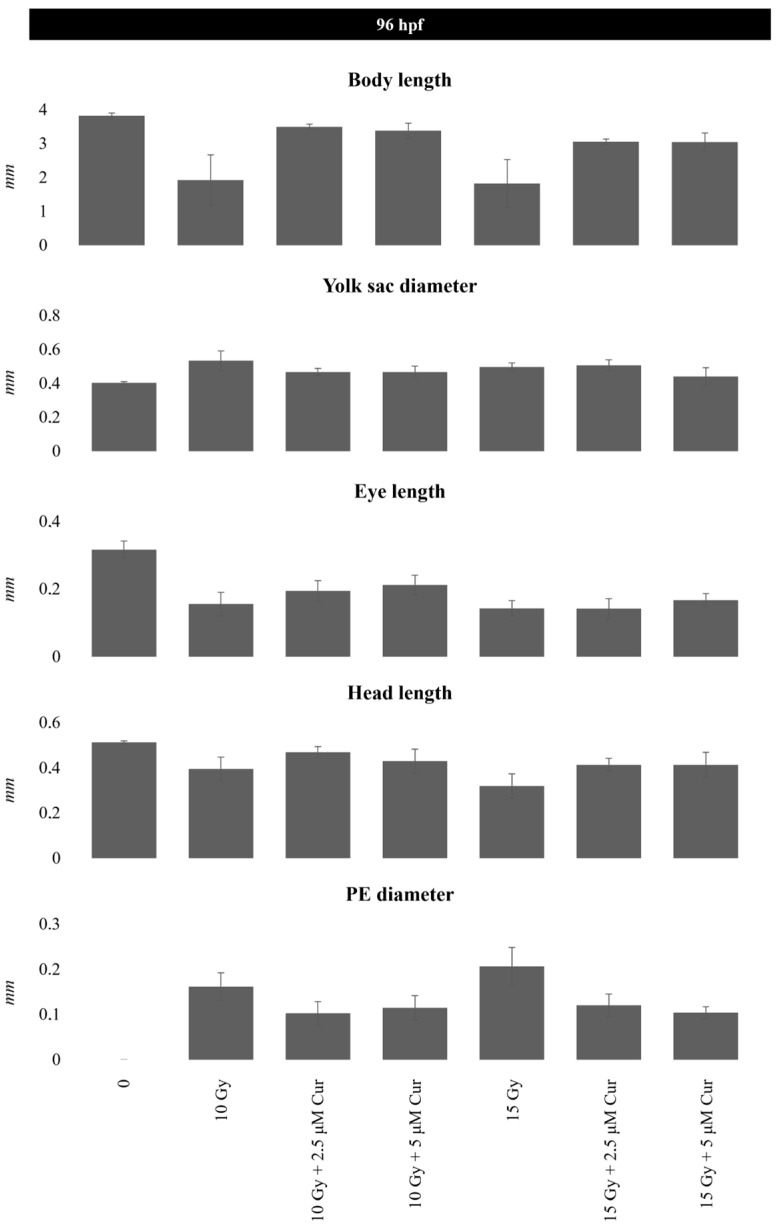
A 96 hpf measurement (mm) of morphological parameters (body length, yolk sac diameter, eye length, head length, and PE diameter) after 10 and 15 Gy of IR treatment, with or without 2.5 and 5 μM curcumin (Cur) pre-treatment. Error bar = ±SD.

**Figure 8:**
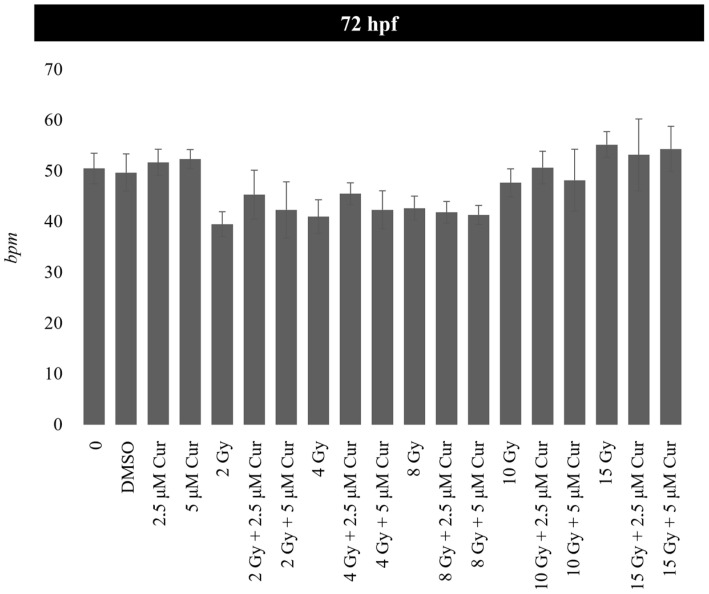
Heart rate values (bpm) of 72 hpf embryos exposed to the experimental doses of 0, 2, 4, 8, and 15 Gy of X-rays in combination with 2.5 or 5 μM curcumin (Cur) pre-treatment. Data are presented as the mean of 3 experiments. Error bar = ±SD.

At the highest dose of 15 Gy, a slight protective effect is exerted up to 96 hpf (87% vs. 91%) (*p*: 0.0658; OR: 0.3187; 95% CI: 0.09897 to 1.026), which disappears at 120 hpf, showing the prevalence of the IR dose effect (Figure 4). Among the malformations, the most important morphological abnormalities, PE and SC, increased in a dose-dependent manner and prevailed for the higher doses (10 and 15 Gy); however, their frequency decreased in the presence of the curcumin pre-treatment up to 96 hpf (Figure S5, Figure 5 and Figure 6). In detail, the SC frequencies at 96 hpf were 55% and 56% in samples treated with 10 and 15 Gy, respectively, vs. 40% and 42% in the 5 µM pre-treated embryos with the same IR doses (*p*_10Gy_ = 0.0472, OR_10Gy_ = 0.5455, 95% CI: 0.3111 to 0.9565; *p*_15Gy_ = 0.0657, OR_15Gy_ = 0.5690, 95% CI = 0.3250 to 0.9962). In addition, a significant reduction in PE is observable at the same time point, as its frequencies were 48% and 70% in samples treated with 10 and 15 Gy, respectively, vs. 25% and 48% in the presence of 5 µM curcumin pre-treatment (*p*_10Gy_ = 0.0012, OR_10Gy_ = 0.3611, 95% CI: 0.1984 to 0.6574; *p*_15Gy_ = 0.0024; OR_15Gy_ = 0.3956, 95% CI: 0.2214 to 0.7069). Furthermore, despite the low frequency of these serious malformations at lower doses, the protective effect of curcumin was also confirmed in these cases. Indeed, the PE frequency at 96 hpf was 11% in 4 Gy-treated embryos and 14.6% in 8 Gy-treated embryos, and it was absent in both the 2.5 and 5 µM curcumin pre-treated specimens. Furthermore, the phenotype severity of a certain malformation was generally mitigated in embryos pre-treated with both the curcumin concentrations, with respect to their gravity, observed in samples subjected to IR treatment alone. Indeed, the evaluation of morphometric parameters at 72 and 96 hpf, such as body length, yolk sac diameter, eye length, and head length, confirmed, to different extents, the protective effect exerted by the curcumin pre-treatment for higher doses of radiation (10 and 15 Gy) (Figure 7). Among these parameters, we focused on the PE diameter, as it is more related to the larvae survival. The PE diameter mean was 0.14 and 0.15 mm at 72 hpf in embryos treated with 10 and 15 Gy and 0.16 and 0.20 mm at 96 hpf with the same doses, respectively. Instead, a significant PE diameter reduction was observed in pre-treated embryos with 2.5 or 5 µM curcumin, as the 72 hpf PE values were 0.085 and 0.01 mm with the dose of 10 Gy (*p*: 0.0014) and 0.067 and 0.11 mm with the dose of 15 Gy (*p*: 0.0127), respectively, while the 96 hpf PE values were 0.08 and 0.12 with the dose of 10 Gy (*p*: 0.0052) and 0.09 and 0.1 with the dose of 15 Gy (*p*: 0.0001) (Figure 7). To quantify the curcumin protection ability, a Protection Rate (PR) has been calculated, as described in the Section 2 Materials and Methods. This calculation showed lower values for the PE diameter and thus better protection in the 72 hpf or 96 hpf specimens subjected to 10 Gy and 2.5 µM curcumin pre-treatment (72 hpf: 39.28%; 96 hpf: 50%), with respect to those treated with 5 µM curcumin (72 hpf: 28.57%; 96 hpf: 25%). Furthermore, the manual evaluation of the heart-beating rate at 72 hpf revealed its decrease in only irradiated embryos compared to controls. Conversely, curcumin pre-treatment exercises a protective role, bringing the heartbeat values closer to those of the controls (Figure 8). Only embryos treated with 15 Gy with or without curcumin suffer from an increased heartbeat, as the PE volume is pronounced and thus the heart tries to compensate, increasing the heart frequency.

The behavioral analysis showed a non-significant increase in the small distance parameter and a decrease in the large distance parameter in the only irradiated embryos. However, the curcumin pre-treatment seems to correct this abnormal larva behavior. For the dose range of 2–10 Gy, a lower increase in the small distance values was observed in pre-treated embryos, with respect to controls (variations from +0.32 to +61.19 mm). A parallel increase in the large distance values was also observed for doses ≥8 Gy, with variations ranging from +6.85 to +10.7 mm.

### 3.4. Gene Expression Analysis of Genes Involved in Oxidative Stress Balance in Response to X-Rays Alone or in Combination 

**Figure 9:**
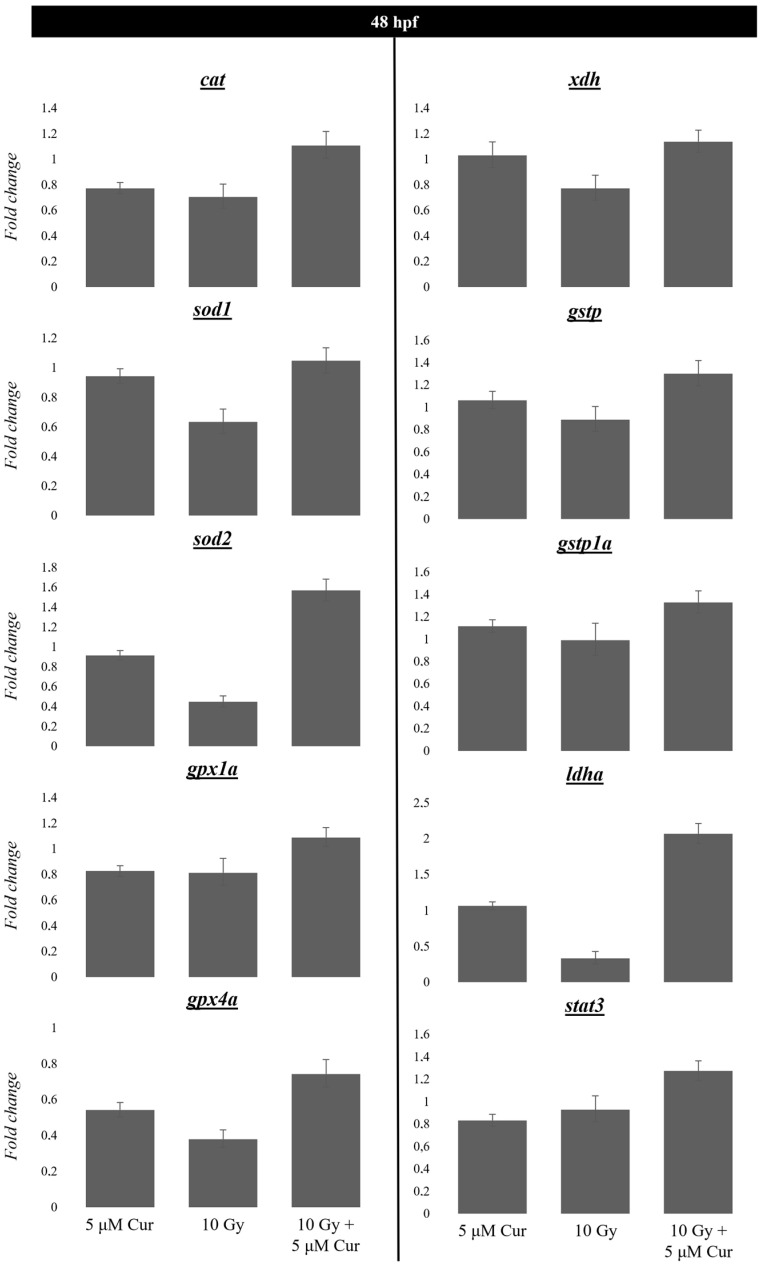
The gene expression variation in targets involved in oxidative stress regulation, in embryos treated with 5 μM curcumin (Cur) and 10 Gy, as single or combined treatments, analyzed by qPCR. Reference sample (Control) = 1. Error bar = ±SD.

Based on the known antioxidant ability of curcumin, we verified by qPCR whether the observed protective effects in sparing embryo malformations and their entities could be due to a variation in the gene expression of enzymes involved in oxidative stress balance: *cat*, *sod1*, *sod2*, *gpx1a*, *gpx4a*, *xdh*, *gstp*, *gstp1a*, *ldha*, and *stat3*. The analysis was conducted at 24 h post-radiation treatment (48 hpf embryos), in embryos treated with 10 Gy and 5 µM curcumin, as these could be supposed to be more stressful treatments. Overall, the results shown in Figure 9 highlight that the 5 µM curcumin treatment alone did not alter the expression levels of these genes by a large amount, with respect to untreated controls. Instead, a more pronounced decrease in their expression levels was observed in embryos subjected to 10 Gy of IR treatment, although their levels were restored to close to those of the untreated controls or even higher in embryos pre-treated with curcumin. In particular, the relative expression level for each gene analyzed in specimens subjected to the combined vs. the IR treatment was as follows: cat: 1.10 vs. 0.70, sod1: 1.04 vs. 0.63, gpx4a: 0.74 vs. 0.37, gpx1a: 1.08 vs. 0.81, xdh: 1.13 vs. 0.77, sod2: 1.56 vs. 0.44, gstp: 1.33 vs. 0.88, gstp1a: 1.32 vs. 0.98, ldha: 2.07 vs. 0.33, and stat3: 1.27 vs. 0.92 (Figure 9).

## 4. Discussion

Natural products are being increasingly valued by the scientific community, due to their potential application as effective, safe, and cheap compounds with radiomodifying roles. Curcumin is a polyphenol derived from the plant *Curcuma longa*, and it exhibits strong antioxidant activity comparable to that of vitamins C and E [46]. It was shown to be a potent scavenger of superoxide anion, hydroxyl, and nitrogen dioxide radicals, which generally deteriorate biomolecules such as proteins, lipids, and nucleic acids [47,48]. In addition, the hydroxyphenyl backbone of curcumin is crucial to its anti-inflammatory activity [49].

The main aim of this research study was to evaluate the curcumin radioprotective effect, using an in vivo approach. In our study, fish embryos were subjected either to a single treatment with curcumin (in the range of 1–10 μM), to X-ray radiation (in the range of 2–15 Gy), or to combined treatments using an 18 h pre-exposure to curcumin (at 2.5 and 5 μM concentrations).

The single treatment with curcumin was carried out by administering the molecule at 6 hpf and then twice a day up to 120 hpf. As also previously reported by other authors, a dose-dependent toxic effect was observed in embryos subjected to continuous curcumin exposure [36,50,51,52,53].

In particular, the concentrations of 7.5 and 10 μM were lethal, leading to 100% mortality at 72 and 48 hpf, respectively. Using 5 μM curcumin, 46% of larvae at 120 hpf died, whereas 47% of alive embryos had malformations, showing early SC and PE manifestations in up to 72% and 52%, respectively. On the other hand, the hatching rate evaluation at 48 hpf showed a delay only for the treatment with 5 μM curcumin, with respect to the control. Similar results were reported by [52], who assessed a significant increase in toxicity after hatching at 48 hpf, due to a major curcumin penetration into embryos after the loss of the protective chorion. Based on our results and the literature data on the protective role of the chorion, we decided to use only the 6 hpf–24 hpf pre-treatment time window, as the safe and low-toxic period for embryo development, to administer curcumin at the concentration of 2.5 and 5 μM, before IR administering. 

As already described by previous research groups, the embryo mortality and morphological aberration rate increased with an increasing radiation dose. However, embryos treated at an advanced embryonic age were less sensitive [34,53]. Indeed, embryos at the stage before the midblastula transition (age < 24 hpf) have not yet fully developed radiation damage repair proteins, resulting in increased radiosensitivity and a lower ability to repair radio-induced damages [54]. This observation justifies our choice of treating embryos with IR at 24 hpf.

In our experiments, the daily assessment of irradiated embryos up to 120 hpf highlighted very low mortality for embryos exposed to 2–15 Gy of X-rays. Indeed, the authors of [32] found an LD50 on the seventh day post-irradiation (dpi) for 24 hpf embryos irradiated with a 20 Gy photon beam. In addition, our experiments showed that IR inflicted malformations in a dose-dependent manner, in accordance with [53], with gross alterations from 8 Gy onwards and frequencies of 50% and 82% in 15 Gy-treated embryos at 48 and 120 hpf, respectively. Particularly, malformations strictly related to survival, such as SC and PE, prevailed for the higher doses (10–15 Gy) and appeared at 48 hpf, with values of 72% and 49%, respectively, at 120 hpf in 15 Gy-irradiated embryos. As suggested by the authors of [31], such radiation-induced malformations show a similarity to those noted in mammals, such as cataract formation and retinal degeneration/atrophy, microcephaly, spinal deformity, or pericardial effusion, confirming the zebrafish embryo model as suitable to test innovative IR therapies.

Moreover, we observed a delayed hatching rate, a reduced heart rate, and impaired swimming behavior with increasing IR doses [55,56,57,58]. In particular, the larvae showed a preference for traveling short distances rather than longer ones, despite a non-significant variation in the average speed. This behavior could be justified by a lower heart rate, mainly associated with small rather than large movements.

After having identified the non-lethal, low-toxic curcumin concentrations and the safer pre-treatment window, as well as explored the toxicity effects caused by the 2–15 Gy of a conventional photon beam, we investigated the curcumin radioprotective role in the combined treatment with IR, using phenotypic and molecular analysis. In our study, we decided to investigate the combination of the lower curcumin concentration (2.5–5 μM) with increasing IR doses, reducing the curcumin pre-treatment to 18 h before the IR administration at 24 hpf.

As expected, considering the low rate of mortality given by the radiation treatment alone, up to 120 hpf, the viability was not significantly affected by the combined pre-treatment with 2.5 and 5 μM of curcumin. Interestingly, a pronounced protective effect was observed as a reduction in the malformed embryos percentage after combined treatments, using both the chosen curcumin concentrations, with respect to the only irradiated embryos. Notably, this effect was visible in the 2–10 Gy range until the last day of observation (120 hpf), and it was greater for the higher curcumin concentration (5 μM), with recoveries of 21% and 11% for doses of 8 and 10 Gy, respectively. On the other hand, the dose of 15 Gy was identified as the threshold dose, as only a slight curcumin protective effect was observed up to 96 hpf (4%), probably due to the greater, irreparable severity of the radiation-induced damage. These results are in line with those reported by the authors of [33], in which a relevant deterioration occurred during day 4 post-IR and increased thereafter at 15–20 Gy radiation doses. The protective effect of curcumin was manifested in the reduced frequency of specific malformations, with SC recoveries of 15% and 14% for the doses of 10 and 15 Gy at 96 hpf, and PE recoveries of 23% and 22% for the same doses at the same time point, respectively. Therefore, the greatest protection, in percentage terms, seems to be exerted on malformations closely related to the embryos and larvae survival, i.e., PE. This acquires greater value considering that severe PE could lead to a circulatory collapse in developing zebrafish and to heart failure in the tardive developmental stages, as described by the authors of [59]. 

Furthermore, curcumin’s protection is exerted not only in terms of PE occurrence but also in terms of reduced damage severity in the pre-treated embryos vs. the irradiated ones. Indeed, the analysis of morphometric parameters at 72 and 96 hpf showed less severe alterations for all the malformations, such as body length, yolk sac diameter, eye length, and head length, although the more interesting recovery was only in the PE diameter.

Thus, in our study, we also quantified the curcumin radioprotective ability by the calculation of a Protection Rate (PR) parameter, which showed that the curcumin pre-treatment at 2.5 μM led to a higher reduction in the PE diameter than obtained at 5 μM, vs. the irradiation alone. As a confirmation of this protection against the heart district, the evaluation of the heart-beating rate at 72 hpf revealed that curcumin pre-treatment brings back the heartbeat values closer to those of the controls for the 2–10 Gy dose ranges using both molecule concentrations. Instead, embryos treated with the dose of 15 Gy, with or without curcumin, suffered from increased heartbeats and more pronounced PE volumes than observed for doses ≤10 Gy. Indeed, the increased heartbeat rate is a physiological mechanism to compensate for a heart suffering due to PE. Thus, in our experiment, the dose of 10 Gy was a threshold dose beyond which the radioprotective role of curcumin was irrelevant.

IR is an important source of exogenous ROS, which in turn, stimulates cellular ROS production and exhausts the tissue’s antioxidant enzymatic system. This mechanism produces structural and molecular damage, causing inflammatory responses and, ultimately, leading to cell death [60,61]. Thus, oxidative stress is a central pathogenic mechanism mediating radiation damage, suggesting that curcumin protection could be associated with an enhanced antioxidant capacity. Consistent with this notion, we observed that the gene expression of some crucial antioxidant enzymes was reduced by irradiation compared to the untreated group, whereas their levels were reported to be close to those of controls or even at higher levels in the pre-treated embryos. In particular, we highlighted the expression levels of *cat*, *sod1*, *sod2*, *gpx4a*, *gpx1a*, *xdh,* and *gstp* genes, involved in the cellular network of oxidative balance maintenance [62].

The O_2_- formed is degraded to H_2_O_2_ by SOD1 in the mitochondrial membrane gap and by SOD2 in the mitochondrial matrix [63,64]. Then, the GPXs (1–4) eliminate H_2_O_2_ in the mitochondrial matrix, by converting tripeptide glutathione (GSH) into oxidized glutathione (GSSG), while uncharged H_2_O_2_ passes through the mitochondrial membrane and is cleared by cytoplasmic SOD1 or CAT. CAT splits H_2_O_2_ into H_2_O and O_2_ and contributes to reducing inflammation and inhibiting caspase-1 activity, IL-1 β production, and maturation [64]. The GSTs exert their detoxification function [65] due to the ability to bind the glutathione (GSH) to the “G” site of GSTp isozymes, playing an important role in the maintenance of the cellular redox state [66]. Instead, the xanthine dehydrogenase (XDH), which normally provides purine biodegradation, can also be converted into XO as a source of free radicals in damaged tissues [67]. Thus, its upregulation is a mechanism for contrasting the XDH depletion in an oxidized environment.

Overall, in our study, the analysis of gene expression levels showed their decrease in the 10 Gy-irradiated embryos, whereas their levels were close to those of controls in embryos subjected to the combined treatment (10 Gy/5 μM curcumin) or in embryos treated with curcumin as a single treatment. In particular, the cat, gpx1, and gpx4a gene expressions also show a 20–50% decrease, induced by curcumin alone. Even in these cases, the two treatments (RI/molecule) show downregulation, and their combination can revert the gene expression, suggesting a synergic action mechanism between the two treatments. Thus, curcumin reverted the downregulation of the *sod1*, *sod2*, *gpx1a/4a*, *cat*, *gstp/1a,* and *xdh* gene expressions, restoring the detoxification capacity of cells subjected to IR treatment.

In addition, another interesting result of our study is the increased expression levels of the transcription factor STAT3 in the combination-treated embryos with respect to the irradiation-treated alone. STAT3 is activated by various growth factors, and it is responsive to the IR stimuli, upregulating a plethora of genes with antioxidant, antiapoptotic, pro-angiogenic, and pro- and anti-inflammatory roles [68,69]. 

In addition, among genes involved in energy metabolism, we investigated the possible variation in *ldha* expression levels. The *a* isoform mainly catalyzes the reaction from pyruvate to lactate by a reaction that oxidizes a NADH molecule [70]. It is one of the main enzymes involved in anaerobic glycolysis, but it can also affect the amount of intracellular oxidative stress [70,71]. We have found a significant increase in its expression levels in the pre-treated embryos compared to the irradiated ones (a 2.07-fold vs. a 0.33-fold change), suggesting that curcumin exerts its protective power against ROS accumulation, also via *ldha* overexpression. 

Given the evidence of an antioxidant action mechanism, major efforts are needed to test the curcumin pre-treatment with radiotherapy schedules, searching for new molecule delivery solutions to overcome the low bio-distribution and bioavailability problems. 

## 5. Conclusions

The IR-induced production of exogenous ROS species accounts for numerous radiotherapy side effects. In zebrafish, the low-toxicity curcumin concentrations of 2.5 and 5 µM produced beneficial effects if administered as an 18 h pre-treatment of photon irradiation using the 2–10 Gy dose range, reducing radiation-induced embryo malformations, both in terms of the frequency and damaged entities. However, using the dose of 15 Gy, these sparing effects disappeared, showing the prevalence of an IR dose effect. In this regard, our group has already planned future experiments to test the combination of curcumin under FLASH irradiation conditions. Indeed, the use of beams releasing the doses at very high dose rates, at least 100 times greater than those used in conventional regimes (FLASH irradiation), represents the new frontier of radiotherapy, with the promising capacity to spare healthy tissues even when subjected to doses higher than those tolerated under conventional dose rates [72]. Thus, we expect that curcumin could show its radioprotective efficacy even over the threshold of 15 Gy, in combination with beams using ultra-high dose rates. 

Overall, gene expression analysis revealed the activation of an antioxidant gene network in pre-treated embryos, as a mechanism sustaining curcumin’s radioprotection ability. IR-induced variation in the transcriptional level of several genes has been frequently associated with changes in chromatin states in a wide variety of cell types and organisms [73,74]. In particular, the IR impact on epigenetic markers such as DNA methylation, histone post-translational modifications, and the relative abundance of microRNA during zebrafish embryogenesis [22,75,76]. From this standpoint, epigenome-wide association studies will be of future interest to help decipher specific epigenetic signatures in the experimental groups of the fish examined in this study.

## Figures and Tables

**Table 1:** A list of primers used for q-PCR.

Target Gene	GenBank^®^ Accession Number	Primer Sequences (5′–3′) Forward (Fw) and Reverse (Rw)	Length (bp)	Annealing T (°C)	Fragment Size (bp)
*cat*	NM_130912.2	F: ATGAAGCCGAGAGAGAGCGT R: TCAGCGTTGTGTTTATCCAGG	20 21	62	154
*sod1*	NM_131294.1	F: AAGAAGCCAGTGAAGGTGACT R: CGTGTCTCACACTATCGGTTG	21 21	62	166
*sod2*	NM_199976.1	F: TGTGCTAACCAAGACCCTTTG R: AACGCTCGCTGACATTCTCC	21 20	62	160
*gpx1a*	NM_001007281.2	F: GCACAACAGTCAGGGATTACA R: AGCCATTTCCAGGACGGAC	21 19	62	165
*gpx4a*	NM_001007282.2	F: TTCACAGCCACAGATATAGATG R: GAAAGCCAGGATGCGTAAACC	22 21	62	171
*xdh*	XM_683891.8	F: ATAGTGATGGATGTGGGCAAG R: TAACCGTCAGGAGAGTAGCG	21 20	62	122
*gstp*	NM_131734.3	F: TCGCAGTCAAAGGCAGATGTG R: GAAACAGCACCAGGTCACCAT	21 21	62	168
*gstp1a*	NM_001020513.1	F: TCTACCAGGAATATGAGACCG R: ACCTTCAGATTCAGCAGCAGA	21 21	62	166
*ldha*	NM_131246.1	F: GTTGGAATGGTTGGAATGGCT R: CTTGTGCGTCTTGAGAAACAG	21 21	62	147
*stat3*	NM_131479.1	F: GGCTGGACAACATTATTGACC R: GGAGGCTTTGGACTCAGGAT	21 20	62	118
*rpl13*	NM_212784.1	F: AGGTGTGAGGGTATCAACATC R: TTGGTTTTGTGTGGAAGCATAC	21 22	62	170

**Table 2:** The percentage (%) of fluorescent signal decay in 24 hpf embryos treated with 5 μM curcumin (Cur) at 6, 9, and 22 hpf and then observed at 24 hpf after the washing-out protocol, by images acquisition every 30–40 min.

Minutes	Fluorescence (%)								
	30	60	90	120	160	200	240	280	320
**Control**	0	0	0	0	0	0	0	0	0
**5 μM Cur**	39.11	29.14	22.71	19.15	14.04	12.44	3.9	1.7	0.2

## Data Availability

The original contributions presented in the study are included in the article/Supplementary Materials, further inquiries can be directed to the corresponding author/s.

## Supplementary Materials

The following supporting information can be downloaded at: https://www.mdpi.com/article/10.3390/antiox13111281/s1. Figure S1: Distribution of normal, malformed and dead embryos treated with 0–10 μM curcumin (Cur); Figure S2: Distribution (%) of the main malformations observed in developing zebrafish embryos exposed 0–10 μM curcumin (Cur); Figure S3: Distribution of normal, malformed and dead embryos treated with 0–15 Gy of X-rays; Figure S4: Distribution (%) of the main malformations observed in developing zebrafish embryos exposed to 0–15 Gy of X-rays; Figure S5: Distribution (%) of the main malformations observed in 48 and 72 hpf zebrafish embryos exposed to curcumin pre-treatment following by X-rays irradiation.
