# Supplementary material for: Curcumin’s Radioprotective Effects on Zebrafish Embryos
Source: Antioxidants (Basel). 2024 Oct 23;13(11):1281. doi: 10.3390/antiox13111281 (PMC11590968; doi:10.3390/antiox13111281)
Supplement: Supplementary file 1 [file antioxidants-13-01281-s001.zip › antioxidants-3202776-supplementary.pdf]

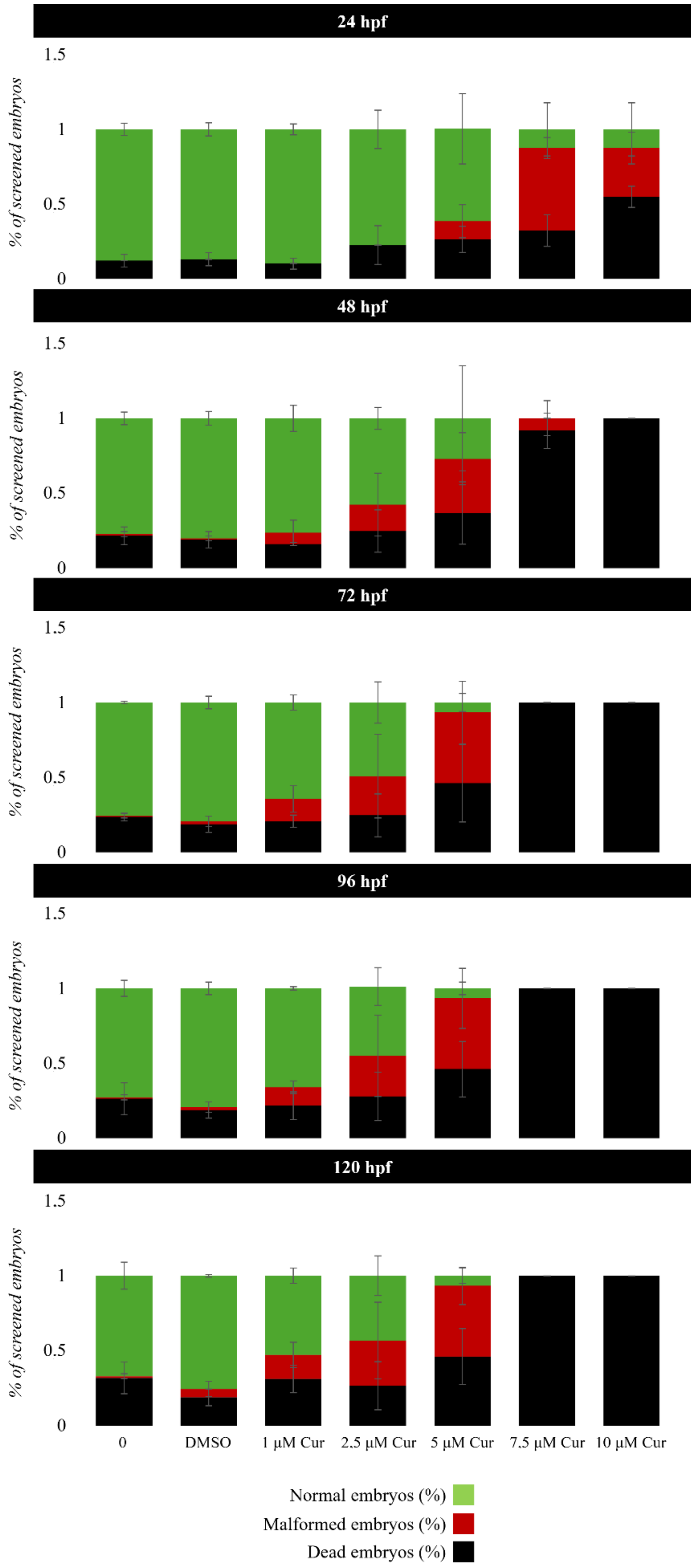

Figure S1: Distribution of normal, malformed and dead embryos treated with 0 – 10 µM curcumin (Cur) Normal (green bar), dead (black bar) and abnormal (red bar) embryos rates in developing zebrafish embryos exposed to 0, 1, 2.5, 5, 7.5 and 10 µM of curcumin. Data are presented as the mean of 3 experiments. Error bar = ± SD.

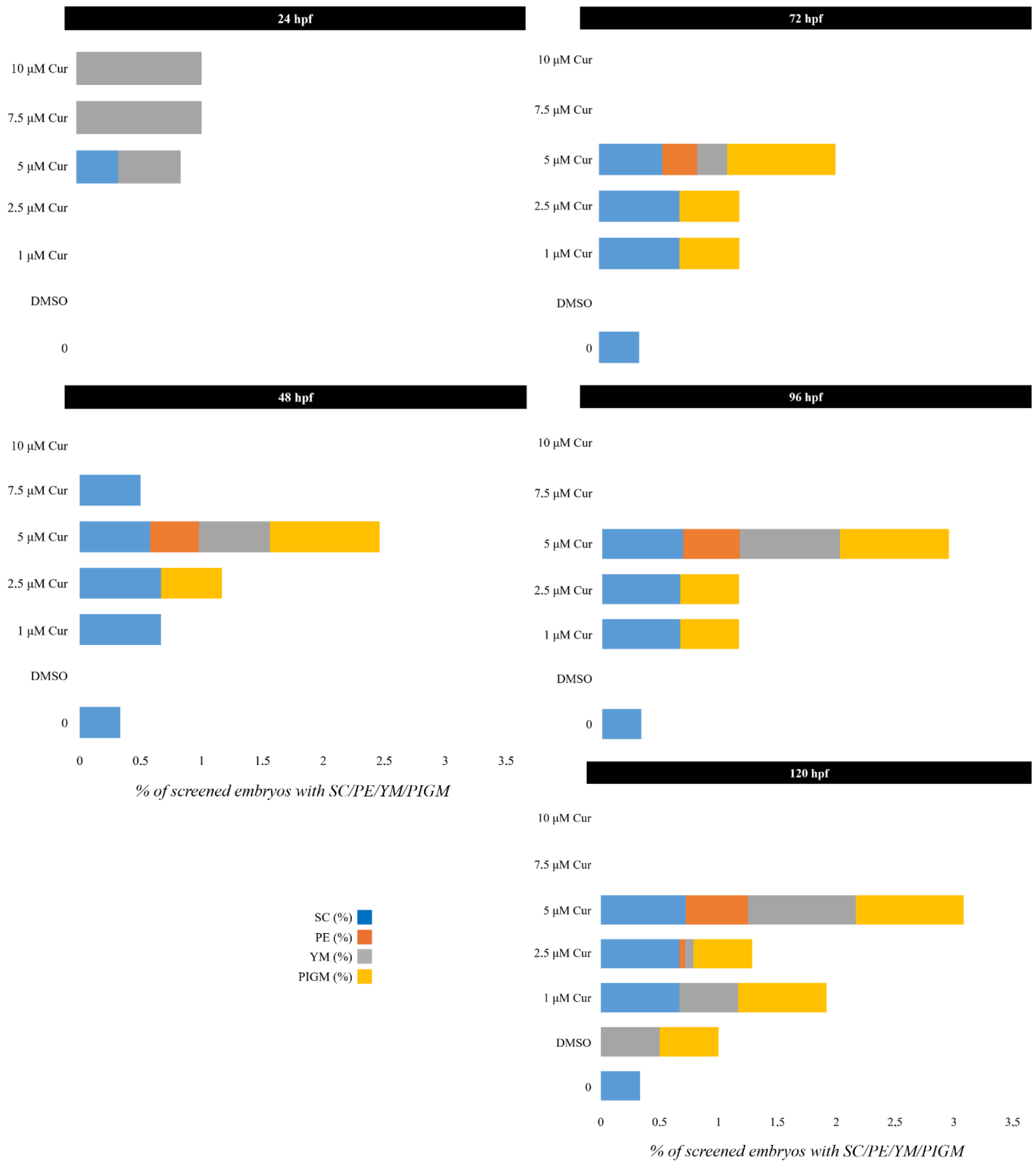

Figure S2 Distribution (%) of the main malformations observed in malformed developing zebrafish embryos exposed to the experimental concentrations of curcumin (Cur): SC (blue bar), PE (orange bar), YM (gray bar), PIGM (yellow bar). Data are presented as the mean of 3 experiments.

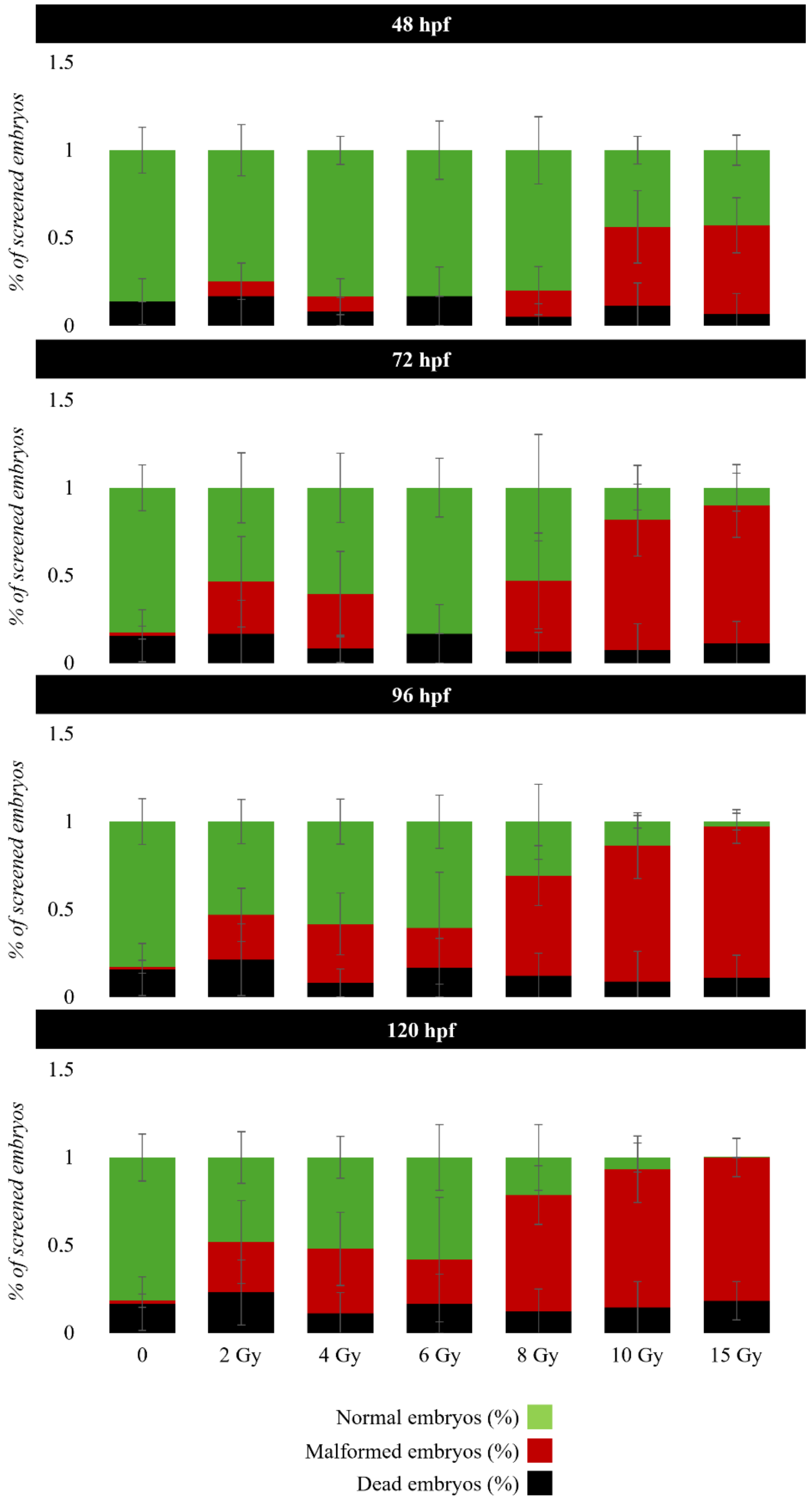

Figure S3: Distribution of normal, malformed and dead embryos treated with 0 – 15 Gy of X- rays. Normal (green bar), dead (black bar) and abnormal (red bar) embryos rates of developing zebrafish embryos exposed to the doses of 0, 2, 4, 6, 8, 10, 15 Gy of X-rays. Data are presented as the mean of 4 experiments. Error bar = ± SD.

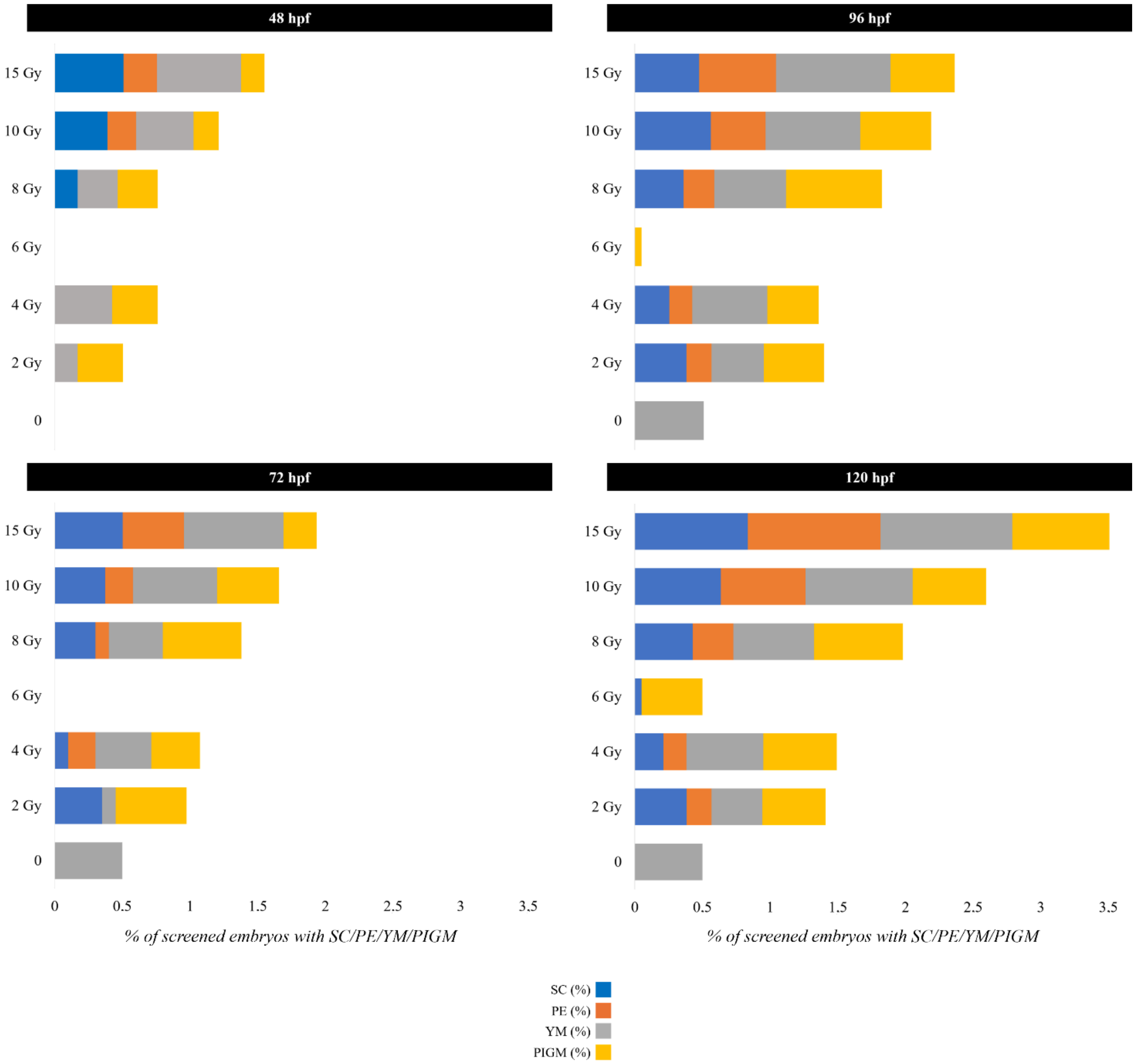

Figure S4 Distribution (%) of the main malformations observed in malformed developing zebrafish embryos exposed to the experimental doses of 0, 2, 4, 6, 8 and 15 Gy of X-rays: SC (blue bar), PE (orange bar), YM (gray bar), PIGM (yellow bar). Data are presented as the mean of 4 experiments.

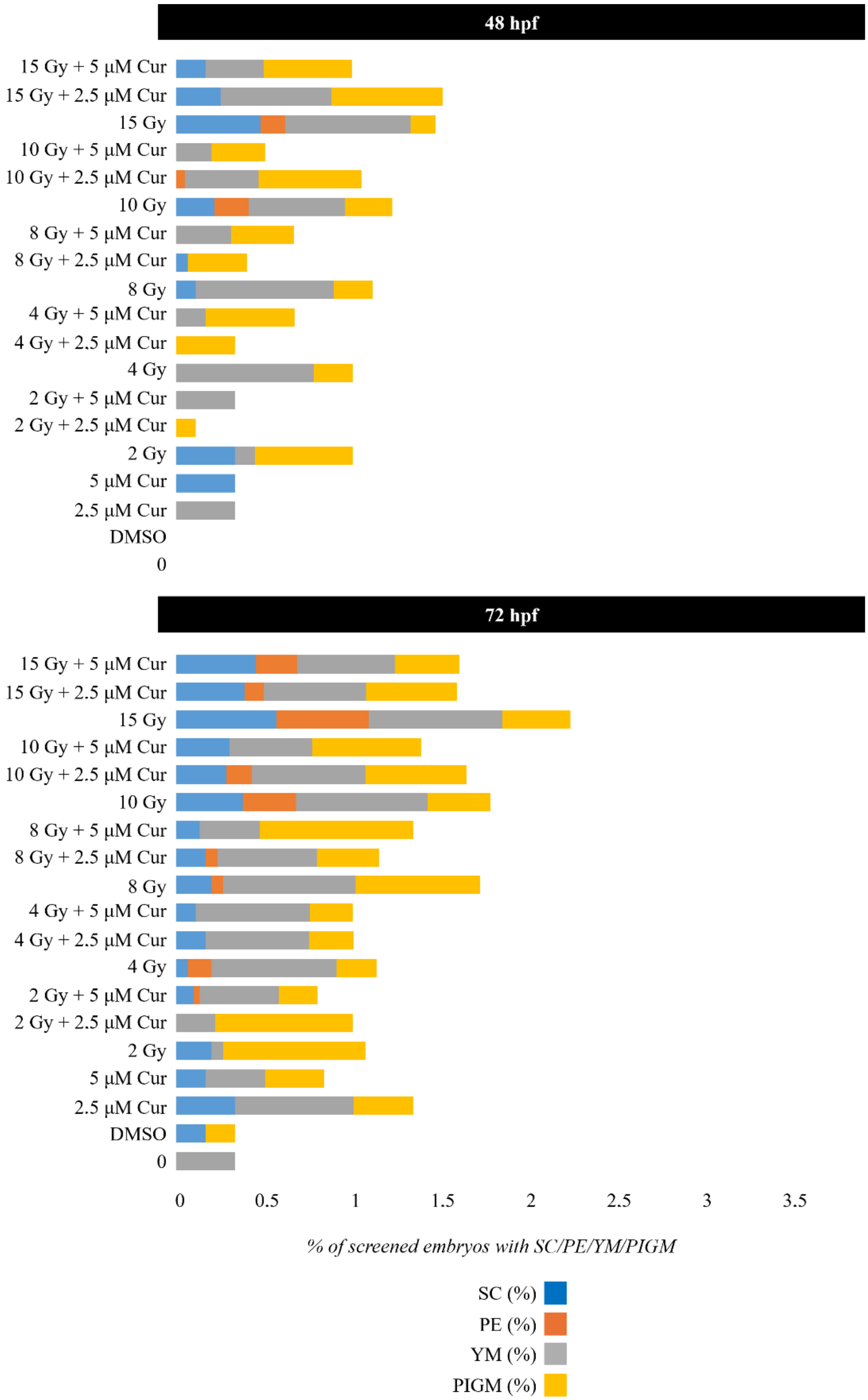

Figure S5 Distribution (%) of the main malformations observed in 48 and 72 hpf malformed zebrafish embryos exposed to a combination of curcumin (Cur) pre-treatment with concentrations of 2.5 or 5 µM, **followed by irradiation** with 0, 2, 4, 8, 10 or 15 Gy of X-rays: SC (blue bar), PE (orange bar), YM (gray bar), PIGM (yellow bar). Data are presented as the mean of 3 experiments.
